# Tyrosine kinases: multifaceted receptors at the intersection of several neurodegenerative disease-associated processes

**DOI:** 10.3389/frdem.2024.1458038

**Published:** 2024-08-16

**Authors:** Max Stevenson, Norah K. Algarzae, Charbel Moussa

**Affiliations:** ^1^The Laboratory for Dementia and Parkinsonism, Translational Neurotherapeutics Program, Department of Neurology, Georgetown University Medical Center, Washington, DC, United States; ^2^Department of Physiology, College of Medicine, King Saud University, Riyadh, Saudi Arabia

**Keywords:** tyrosine kinase, neurodegenerative diseases, c-KIT, autophagy, inflammation, blood vessels

## Abstract

Tyrosine kinases (TKs) are catalytic enzymes activated by auto-phosphorylation that function by phosphorylating tyrosine residues on downstream substrates. Tyrosine kinase inhibitors (TKIs) have been heavily exploited as cancer therapeutics, primarily due to their role in autophagy, blood vessel remodeling and inflammation. This suggests tyrosine kinase inhibition as an appealing therapeutic target for exploiting convergent mechanisms across several neurodegenerative disease (NDD) pathologies. The overlapping mechanisms of action between neurodegeneration and cancer suggest that TKIs may play a pivotal role in attenuating neurodegenerative processes, including degradation of misfolded or toxic proteins, reduction of inflammation and prevention of fibrotic events of blood vessels in the brain. In this review, we will discuss the distinct roles that select TKs have been shown to play in various disease-associated processes, as well as identify TKs that have been explored as targets for therapeutic intervention and associated pharmacological agents being investigated as treatments for NDDs.

## 1 Introduction

Tyrosine kinases (TKs) are catalytic enzymes that function by phosphorylating tyrosine residues on downstream substrates as a means of signal transduction. They can exist as either membrane bound receptor tyrosine kinases (RTKs), where they dimerize and auto-phosphorylate to promote transmembrane signaling, or in the cytoplasm, where they can interact with other proteins within the cell. There are over 120 known TKs that are expressed on and within a wide variety of cell subtypes throughout the mammalian body and subsequently, are associated with an array of different processes: these include cellular development and differentiation, proliferation, metabolism, mobilization, and production of key cellular substrates (Schlessinger, 2000; Lemmon and Schlessinger, 2010). TK overexpression has been observed in various cancers, consistent with the finding that dysregulated TK signaling has been shown to promote unrestrained cell growth (Krause and Van Etten, 2005; Jiang and Ji, 2019). As such, inhibition of TKs has been explored as a potential medium for treating cancer: TKIs such as nilotinib, imatinib, and dasatinib, etc. have been utilized to treat various cancers by blocking TK-mediated cellular proliferation, primarily due to their ability to induce autophagy-dependent apoptosis of cancer cells (Nam et al., 2005; Yu et al., 2013; Iqbal and Iqbal, 2014). While the functional capacity of TK signaling is indeed vast, it has been associated with dysregulated processes associated with a host of NDDs, including protein dyshomeostasis, inflammation, neurotransmitter imbalance, oxidative stress, and blood vessel damage (Ohnishi et al., 2001; Gilfillan and Rivera, 2009; Dorison et al., 2017; Gómez-Virgilio et al., 2022).

## 2 Tyrosine kinase functions in neurodegenerative disease

### 2.1 Tyrosine kinases in autophagy

TK signaling plays a fundamental role in autophagy and it is associated with cellular survival and proliferation, as several TKs activate the PI3K-Akt-mTOR pathway (Renné et al., 2007; Ebi et al., 2011; Jiang and Ji, 2019), which is responsible for, among other processes, regulation of protein and lipid synthesis, RNA translation, and cellular metabolism (Davis et al., 2015; Fruman et al., 2017). Autophagy is the catabolic process by which cells collect and degrade unwanted or unnecessary components, and mTOR signaling is known to negatively regulate autophagy, identifying mTOR-converging pathways as greatly important for restraining cell growth, and promoting autophagy (Pattingre et al., 2008; Jian et al., 2019; Wang and Zhang, 2019). With regard to NDDs, it is well-documented that impaired autophagy is associated with neurotoxic protein aggregation in the brains of patients suffering from proteinopathies, a subset of NDDs defined by toxic protein inclusions (Nixon et al., 2005; Hebron et al., 2013b; Salminen et al., 2013; Lee et al., 2022). Undegraded autophagic vacuoles have been observed in the neurites of patients with Alzheimer's Disease (AD) (Boland et al., 2008), while upregulation of several tyrosine kinases have been detected in the hippocampi of postmortem AD patients and the substantia nigra of Parkinson's Disease (PD) patients, regions known to be vulnerable to pathological protein aggregation in these diseases (Fowler et al., 2019), implicating TK signaling in protein accumulation and disease pathogenesis, although the distinct mechanisms of action remain unclear. Additionally, both pharmacological inhibition and genetic ablation of various TKs including Abelson (Abl), platelet-derived growth factor receptors (PDGFR)α, and fused-in sarcoma (Src) have been shown to reduce concentrations of amyloid-beta (Aβ) and phospho-tau (pTau) in preclinical models of AD and PD (Dhawan and Combs, 2012; Javidnia et al., 2017; Fowler et al., 2019). PIKFYVE is involved in several neurological diseases and it plays an important role in maintenance of lysosomal functioning, endosomal trafficking, and autophagy (Rivero-Ríos and Weisman, 2022), and was recently shown to be a relevant target to alleviate ALS pathology (Crunkhorn, 2023; Hung et al., 2023). PIKFYVE and PIP5K1A are enzymes targeted as therapies for cancer, as their inhibition has been shown to induce autophagy (Lee et al., 2013; O'Connell and Vassilev, 2021; Zhang Z. et al., 2021; González-Rodríguez et al., 2022). Stimulation of autophagy may prevent intracellular protein accumulation, such as the classical α-synuclein (α-syn), p-tau and Aβ and extracellular plaque deposits (Giannakopoulos et al., 2003; Ethell, 2010) in several models of AD and PD (Khandelwal et al., 2011; Lonskaya et al., 2013a,b, 2014). TKIs stimulate clearance of intracellular neurotoxic proteins via autophagy (Hebron et al., 2013a; Lonskaya et al., 2013a, 2014; Fowler et al., 2019; La Barbera et al., 2021). Subsequent clinical trials using TKIs to treat AD and PD patients are underway to determine if stimulation of autophagy is sufficient to improve cognitive outcomes in human patients (Pagan et al., 2020; Turner et al., 2020). Collectively, these data suggest that multi-kinase inhibition provides an effective means of alleviating neurodegenerative pathologies via induction of autophagic clearance of neurotoxic protein aggregates (Hebron et al., 2013b; Mahul-Mellier et al., 2014; Lonskaya et al., 2015; Javidnia et al., 2017; Fowler et al., 2019).

### 2.2 Tyrosine kinases in neuroinflammation

Several downstream substrates of TK signaling are also implicated with various inflammatory signaling pathways, identifying TK inhibition as a potential means of reducing neuroinflammation in NDDs as well. Senescent glial cells, including astrocytes and microglia, have been identified as primary contributors to neurodegeneration, in part due to their propensity to express a senescence-associated secretory phenotype (SASP) by which they secrete pro-inflammatory molecules that can damage surrounding tissue (Martínez-Cué and Rueda, 2020; Di Micco et al., 2021; Rachmian et al., 2024; Rim et al., 2024). Subsequently, identification of the role of TK signaling in senescent cells represents a milestone toward halting neuroinflammation in neurodegenerative disorders. A primary inflammatory substrate of several RTKs is Janus kinase (JAK), a kinase that when phosphorylated by upstream TKs can activate signal transducer and activator of transcription (STAT) proteins, transcription factors that regulate expression of several inflammatory genes including interferons, interleukins, and chemokines as a means of promoting inflammation (DeBerry et al., 1997; Rawlings et al., 2004; Hu X. et al., 2021). TK signaling can also lead to activation of nuclear factor kappa-light-chain-enhancer of activated B cells (NFκB), another type of transcription factor that is highly involved in regulation of both innate and adaptive immune responses in various cell subtypes, including T cells, B cells, macrophage, monocytes, dendritic cells, and neutrophils (Natarajan et al., 1998; Kumar et al., 2004; Perkins, 2007). Additionally, TK activity has been linked to activation of nuclear factor of activated T-cells (NFAT), a series of transcription factors expressed in most immune cells that can further contribute to the production of inflammatory cytokines and chemokines and have been implicated in several inflammatory disorders, including multiple sclerosis (MS) (Ghosh et al., 2010; Lodygin et al., 2013; Baer et al., 2017). c-Abl, c-KIT, and FYN, reduce microglial activation and tau phosphorylation, and have been investigated as therapeutic targets (Dhawan and Combs, 2012; Hebron et al., 2013b; Mahul-Mellier et al., 2014; Nygaard et al., 2014). Finally, TK signaling can activate downstream interferon-regulatory factors (IRFs), a family of transcription factors that regulate the expression of interferons and play a vital role in the maturation of various immune cells, including macrophage and lymphocytes (Kierdorf et al., 2013; Li et al., 2014; Negishi et al., 2018; Luo et al., 2019). Considering the multiple downstream inflammatory targets of TK signaling, TK inhibition thus represents a method for blocking these pathways and mitigating rampant inflammation as a means of reducing NDD pathology.

### 2.3 Tyrosine kinases and neurotransmitters

Previous findings have demonstrated dysregulated levels of various neurotransmitters across several NDDs: these include reduced dopamine levels in PD (Lotharius and Brundin, 2002; Masato et al., 2019), reduced dopamine and acetylcholine levels in AD (Lombardo and Maskos, 2015; Nobili et al., 2017; La Barbera et al., 2022), increased glutamate levels in MS and amyotrophic lateral sclerosis (ALS) (Plaitakis and Caroscio, 1987; Macrez et al., 2016), and decreased gamma-aminobutyric acid (GABA) levels in epilepsy and Huntington's Disease (HD) (Czuczwar and Patsalos, 2001; Garret et al., 2018). Interestingly, several TKs have been found to be upstream of various processes regulating neurotransmitter activity, production, and breakdown. Clinical trials using nilotinib, a TKI previously used as a treatment for chronic myelogenous leukemia, identified significantly altered microRNAs (miRNAs) that targeted various neurotransmitter-associated genes, including catechol-O-methyltransferase (COMT), aldehyde dehydrogenases (ALDHs), and sulferotransferases (SULTs) (Anderson et al., 2022; Stevenson et al., 2023). TKs have also been shown to phosphorylate certain neurotransmitter receptors, including N-methyl-D-aspartate (NMDA) glutamatergic receptors and GABA receptors, and can also control neurotransmitter release into the synapse (Moss et al., 1995; Ohnishi et al., 2001; Trepanier et al., 2012; Liu et al., 2019; Rajani et al., 2021). Some TKs have also been found to mediate regulators of tyrosine hydroxylase (TH) (Fujisawa and Okuno, 2005; Stevenson et al., 2023), an enzyme that catalyzes the production of the dopamine precursor L-DOPA and whose activity serves as the rate-limiting step of dopamine production. Additional downstream targets of TK signaling associated with neurotransmitter regulation include aldehyde dehydrogenase, SLC18A3 (the vesicular acetylcholine transporter), and spermine oxidase (SMOX), further identifying potential mechanisms by which TKs can potentially regulate neurotransmitter levels (Chaturvedi et al., 2014; Stevenson et al., 2023).

### 2.4 Tyrosine kinases and blood vessels

**Figure 1:**
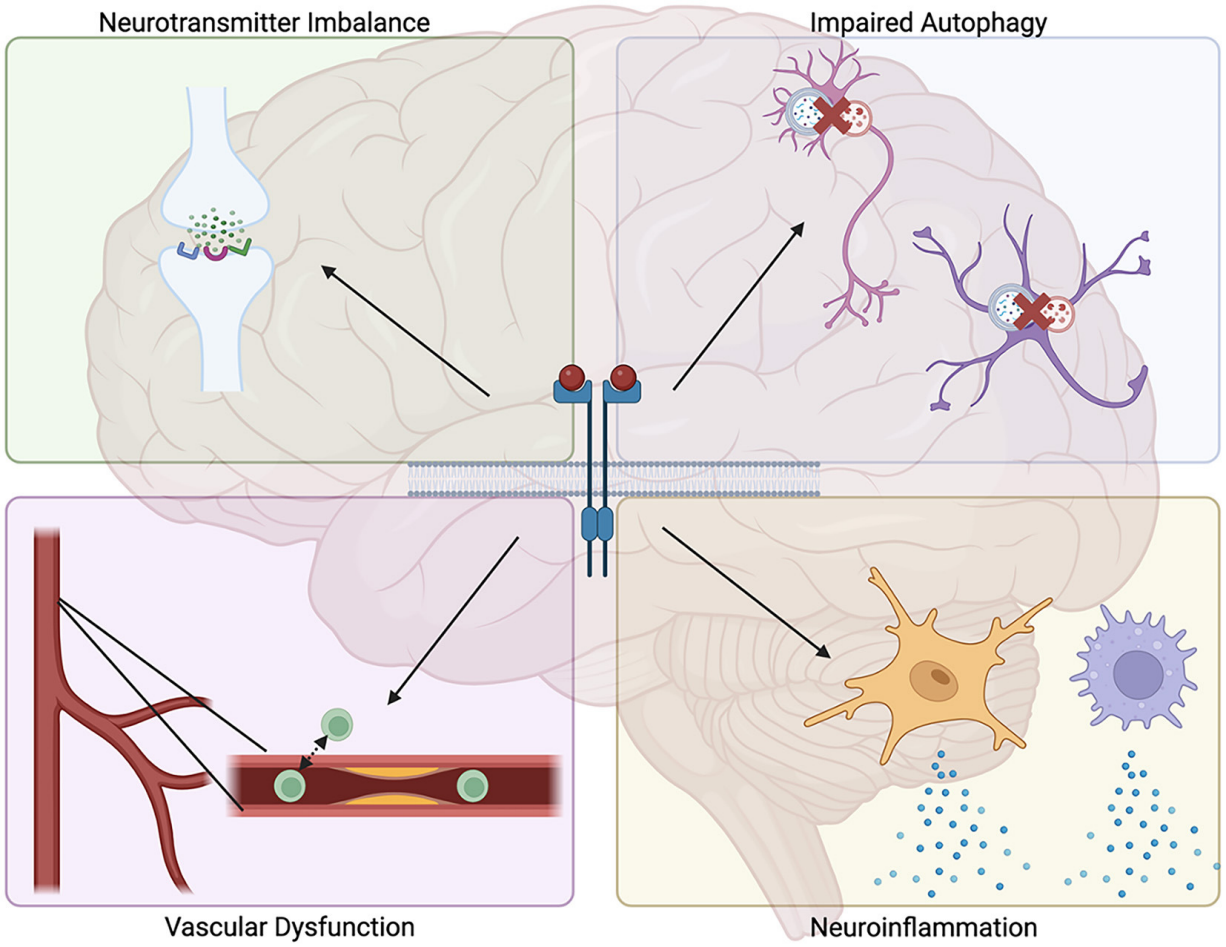
Proposed mechanisms for how tyrosine kinase signaling may contribute to neurodegenerative disease pathology. Tyrosine kinase signaling has been demonstrated to promote several processes associated with neurodegenerative diseases, including impaired autophagic clearance of neurotoxic protein aggregates, increased neuroinflammation, increased vascular fibrosis and permeability, and dysregulated neurotransmitter levels.

Damage to blood vessels is another feature prevalent in certain NDDs. In particular, AD displays numerous vascular deficits, including vascular fibrosis and calcification, Aβ deposition along brain vasculature, and increased blood vessel permeability, which are associated with increased rates of cognitive decline (Wegiel et al., 2002; Davis et al., 2004; Iturria-Medina et al., 2016; Bennett et al., 2018; Govindpani et al., 2019). Indeed, impaired cerebral blood flow has been demonstrated to be a primary contributor to disease onset in AD, highlighting the significant role of vascular integrity in brain health (Roher et al., 2004; Ristori et al., 2020; Swinford et al., 2023). Decreased cerebral blood flow and blood-brain barrier dysfunction have also been demonstrated in other NDDs, including PD, HD, ALS, and MS, suggesting convergent pathogenic mechanisms by which these disorders may arise (Lim et al., 2017; Fowler et al., 2021; Nishihara et al., 2022; Steinruecke et al., 2023). Signaling via TKs expressed on blood vessel endothelial cells has been implicated in features that contribute to impaired blood vessel health: these include formation of atherosclerotic plaques, accumulation of macrophages, matrix metalloprotease (MMP) production, and fibrotic thickening of blood vessels (Guerrot et al., 2011; Zhu et al., 2015; Jian et al., 2019). Additionally, TK inhibition has been shown to reduce many of these features and attenuate the inflammatory response, resulting in improved vascular health (Hebron et al., 2017; Fowler et al., 2019; Stevenson et al., 2023). Certain families of TKs have also been shown to regulate angiogenesis, a process that while primarily linked to tumor growth and metastasis has also been implicated in the progression of PD (Hicklin and Ellis, 2005; Fowler et al., 2021). Ultimately, these findings identify TKs as regulators of several functions believed to contribute to NDD pathogenesis, suggesting them as potential targets for therapeutic intervention when treating these disorders (Figure 1).

## 3 Distinct tyrosine kinases associated with disease pathology

### 3.1 c-KIT and mast cells in neurodegenerative pathologies

c-KIT (CD117) is an RTK that consists of an extracellular domain, a transmembrane domain, a juxtamembrane domain, and a kinase domain. Its natural ligand is a growth factor known as stem cell factor (SCF) and it is primarily expressed on hematopoietic stem cell (HSC)-derived cells such as mast cells, the innate immune cells primarily responsible for responding to allergens (Galli et al., 1993; Pittoni et al., 2011). c-KIT signaling has been shown to be vital for both the mobilization of HSC-derived progenitors from the bone marrow to peripheral tissues, which direct these progenitors via SCF gradients, as well their development into mature cells such as mast cells and basophils. c-KIT over activation leads to atypical mast cell proliferation, maturation and degranulation (Sevilla and Grichnik, 2024), which results in detrimental inflammatory processes such as release of pro-inflammatory factors (Jensen et al., 2008; Yu et al., 2023). In both the mouse and human, bone marrow mast cell progenitors are released into the bloodstream where they subsequently migrate into peripheral tissues, during which time they mature and become terminally differentiated under the influence of cytokines within the surrounding milieu (Metcalfe et al., 1997; Dahlin and Hallgren, 2015). c-KIT signaling is also involved in amyloid precursor protein (APP) phosphorylation and Aβ production (Chen et al., 2019). Interestingly, c-KIT has been shown to be expressed in the brain on neurons, astrocytes, and microglia, mainly during neural development in regions including the cerebellum, hypothalamus, and hippocampus, before tapering off post-development (Zhang and Fedoroff, 1997). However, c-KIT expression has been detected on microglia in the brains of transgenic AD mice (Stevenson et al., 2024), consistent with findings that it is upregulated following brain injury (Zhang and Fedoroff, 1999) and indicating a potential role for c-KIT in neurodegeneration in regions vulnerable to disease pathology.

The downstream substrates of c-KIT include PI3K, whose phosphorylation can result in activation of mTOR, leading to subsequent proliferative signaling and decreased autophagy, JAK, which is responsible for initiation of the JAK-STAT inflammatory signaling cascade (DeBerry et al., 1997; Lennartsson and Rönnstrand, 2012), and Src (Lennartsson et al., 1999), a tyrosine kinase capable of phosphorylating several intracellular substrates including STATs, tau and NMDA receptors, among others. These substrates implicate c-KIT in various processes involved with NDDs such as autophagy, inflammation, and synaptic dysfunction (Ali and Salter, 2001; Hu X. et al., 2021; Kma and Baruah, 2022). Regarding mast cells, the primary cells that express c-KIT, c-KIT is known to be integral to their development from immature mast cells to mature mast cells but has also been shown to cross-link with FcεRI, the primary mast cell receptor responsible for responding to allergens, to enhance the allergic response. Considering the emerging role of mast cells in NDDs such as AD and MS (Vermersch et al., 2022; Dubois et al., 2023), inhibiting c-KIT signaling on mast cells thus represents a potential mechanism for reducing inflammation in these disorders. Together, these findings suggest c-KIT as a convergent target for stimulating autophagic clearance of neurotoxic protein aggregates as well as mitigating both CNS neuroinflammation and mast cell-mediated inflammation. The functional involvement of peripheral mast cells in inflammatory conditions is well established. Increasing evidence indicate that in the brain, inflammation is strongly involved in the pathogenesis of NDDs (Kempuraj et al., 2017, 2019; Traina, 2017).

Human c-KIT has evolved as a hot therapeutic target for several NDDs based on evidence of efficacy in animal models and human disease (Dubreuil et al., 2009; Piette et al., 2011; Li et al., 2020; Stevenson et al., 2024). c-KIT signaling is significantly increased in postmortem brains of AD and PD patients, while a recent report showed a pathogenic role for c-KIT-expressing mast cells mediating inflammation and neuromuscular junction denervation in muscles from SOD1G93A ALS rats (Trias et al., 2018). Mast cells and microglia orchestrate immune responses in AD and PD, suggesting targeting of mast cells as a neuroprotective strategy (Sandhu and Kulka, 2021). Microglia and mast cell activation is reported in MPTP-treated models of PD (Selvakumar et al., 2020), while increased mast cells were also observed in PD models (Zhang X. et al., 2021). In ALS, the inflammatory mechanisms in peripheral motor axon degeneration are attributed to mast cells and neutrophils (Trias et al., 2018), and c-KIT inhibition results in reduced pathology in TDP43 models of ALS (Spiller et al., 2018). Imatinib, a c-KIT inhibitor that also inhibits Abl and PDGFRα (Iqbal and Iqbal, 2014), has been shown in preclinical models of AD to decrease levels of Aβ and pTau (Weintraub et al., 2013; Gardner et al., 2016), while it has also displayed efficacy at reducing CNS inflammation and demyelination in MS models (Azizi et al., 2014) and dopaminergic neuron death in PD models (Imam et al., 2011), indicating its potential as a therapeutic for NDDs. Pharmacologically targeting c-KIT provides a golden opportunity to investigate the differential effects of these compounds in AD, PD, ALS, and frontotemporal dementia (FTD), as well as mast cell disease with c-KIT mutations (mastocystosis) and without (psoriasis and urticaria) c-KIT mutations. Masitinib is a c-KIT/PDGFR inhibitor currently under clinical investigation as a potential target of cell-signaling pathways associated with neurodegeneration, including neuroinflammation, excitotoxicity, and cognitive improvement in AD (Dubreuil et al., 2009; Folch et al., 2015; Fagiani et al., 2020; Dubois et al., 2023). Patients with mild-to-moderate AD who received 4.5 mg/kg masitinib daily (NCT00976118 and NCT01872598) displayed significant improvement on cognitive measures compared to placebo in a Phase III clinical trial (Dubois et al., 2023). Masitinib is also in late-stage clinical trials for multiple sclerosis (NCT01450488 and NCT01433497); in a Phase III clinical trial, patients who received 4.5mg/kg masitinib daily displayed significant benefit vs. placebo (Vermersch et al., 2022). Subsequent confirmatory studies are underway (NCT05441488) to establish masitinib as an effective treatment for mast cell-mediated pathology across NDDs. However, masitinib's efficacy may be limited to c-KIT effects on neuroinflammation and may not be mediated by multi-kinase inhibition that simultaneously underlies autophagy and neuroinflammation, and there is no evidence that masitinib reduces toxic proteins. Masitinib is also in late-stage clinical trials for multiple sclerosis (Arsenault et al., 2022; Vermersch et al., 2022).

### 3.2 Discoidin domain receptor-1

DDR1 is a TK that can be expressed as a receptor or in the cytoplasm. The natural ligand for DDR1 is collagen (both fibrillar and non-fibrillar) and it is predominantly expressed on epithelial cells, including those along the vasculature. DDR1 can also be expressed on CNS cells (Vogel et al., 2000; Vilella et al., 2019), and its increased expression has been associated with various types of cancers, suggesting a role for DDR1 in cellular proliferation. DDR1 activation leads to a host of cellular responses, including microglial activation, accumulation of macrophages, and production of matrix metalloproteases (MMPs) that can damage the blood-brain barrier (BBB) (Seo et al., 2008; Franco et al., 2009; Zhu et al., 2015). Also downstream of DDR1 activation is production of collagen, a primary component of fibrotic plaques (Nadkarni et al., 2009). Indeed, DDR1 has been implicated in several fibrotic disorders, further suggesting its role in both vascular damage and neuroinflammation in NDDs (Franco et al., 2008; Borza et al., 2017). It has been previously shown that DDR1 is upregulated in postmortem AD and PD brains, and that lentiviral shRNA knockdown of DDR1 reduces the levels of α-syn, tau, and Aβ and prevents cell loss *in vivo* and *in vitro* (Hebron et al., 2013b; Fowler et al., 2019). DDR1 knockdown also alters brain immunity and significantly reduces the level of triggering receptor expressed on myeloid cells (TREM)-2 and microglia, suggesting that DDR1 inhibition is a potential target to clear neurotoxic proteins and reduce inflammation and vascular fibrosis in neurodegeneration (Hebron et al., 2017; Wang et al., 2020; Rachmian et al., 2024). Nilotinib is a potent DDR1 inhibitor with evidence from several clinical investigations for NDDs, including AD, PD, and HD (Pagan et al., 2020; Turner et al., 2020; Fowler et al., 2021; Anderson et al., 2022; Stevenson et al., 2023), showing nilotinib effects on autophagy, blood vessels and inflammation and subsequent effects on cognition and long-term motor stabilization. Evaluation of safety and tolerability of 150 mg nilotinib in HD patients revealed alterations to CSF miRNAs targeting autophagy, neurotransmitter regulation, and glial activity, suggesting alleviation of pathological mechanisms in HD patients, although no behavioral changes were detected (Anderson et al., 2022). A Phase II clinical trial in PD patients receiving either 150 mg or 300 mg nilotinib also revealed relevant biomarker changes, including increased CSF homovanillic acid (HVA) levels and reduced α-syn and pTau, compared to placebo, suggesting therapeutic benefit and indicating the need for further investigation via late-stage clinical trials (Pagan et al., 2020). Lastly, a Phase II clinical trial in mild-to-moderate AD patients receiving either 150 mg or 300 mg nilotinib revealed decreases in amyloid PET signal and slowed hippocampal volume loss, as well as reductions in CSF Aβ_40_ and Aβ_42_ and alterations in CSF miRNAs associated with autophagy, inflammation, and vascular fibrosis (Turner et al., 2020; Stevenson et al., 2023). Together, these clinical data suggest DDR1 inhibition as another potential therapeutic medium for treating NDDs.

### 3.3 Src family kinases in neurodegeneration

Src kinases are a family of non-receptor tyrosine kinases involved in a variety of signal transduction pathways associated with NDD pathogenesis. Structurally, Src kinases possess an N-terminal segment, SH3 and SH2 domains that are activated by other protein kinases (including tyrosine kinases), a linker segment, a tyrosine kinase domain, and a C-terminal tail (Salter and Kalia, 2004). As such, Src kinases serve as integral mediators of intracellular tyrosine kinase signaling cascades. Additional upstream signaling pathways shown to converge on Src include G-protein coupled receptors (GPCRs), various cytokine and chemokine receptors, and Ras, the latter of which suggests a role by which Src inhibition may induce autophagy (Parsons and Parsons, 2004; Matozaki et al., 2021). Activated Src has been found to phosphorylate tau at tyrosine residues, implicating its action is tau pathology, while Src can also phosphorylate NMDA receptors, thereby controlling their expression at the postsynaptic density and implicating Src in disease pathogenesis in such disorders as AD and HD (Ali and Salter, 2001; Salter and Kalia, 2004; Scales et al., 2011). Src inhibition has been shown to attenuate microglial reactivity and associated inflammation, suggesting Src may also have a role in neuroinflammatory signaling in NDDs (Dhawan and Combs, 2012). It was previously demonstrated that Src inhibition reduced microglial activation and subsequent inflammation, resulting in greater numbers of dopaminergic neurons and improved behavior in a mouse model of PD (Tai et al., 2013). Ultimately, the multifaceted nature of Src signaling makes it an intriguing target for inhibiting numerous processes associated with NDD pathology. We recently showed that Src inhibition with the Abl/Src inhibitor bosutinib reduces dopamine catabolism and α-syn levels in patients with dementia with Lewy bodies and significantly improves activities of daily living (Pagan et al., 2022). Bosutinib has also been found to improve memory in an open label study in mixed dementia patients (Mahdavi et al., 2021) Fyn is another member of the Src kinase family that has been shown to contribute to NDD processes. Known functions associated with Fyn include leukocyte signaling, cell adhesion, and synaptic modeling (Calautti et al., 2002; Parravicini et al., 2002). Specifically, Fyn is a Src kinase that has been demonstrated to phosphorylate NMDA receptors and control their trafficking at the synapse, suggesting a role for Fyn in impaired long-term potentiation and glutamate homeostasis (Trepanier et al., 2012). Fyn expression has been shown to be increased in a subset of neurons from AD brains, which also displayed increased levels of abnormally phosphorylated tau. Additionally, Fyn has been shown to phosphorylate APP and generate toxic Aβ species, leading to neuron death (Shirazi and Wood, 1993; Nygaard et al., 2014; Iannuzzi et al., 2020). These interactions with both Aβ and tau suggest Fyn as a viable therapeutic target for alleviating AD pathologies. Furthermore, Fyn has been found to modulate transcriptional upregulation and posttranslational modification of the microglial Kv1.3 potassium channel in models of PD, resulting in an augmented neuroinflammatory response and signifying a means by which Fyn activity may be involved with disease-associated inflammation (Sarkar et al., 2020).

### 3.4 Abelson (c-Abl) in neurodegeneration

c-Abl is a specific member of the Src kinase family that has been widely implicated in various NDDs (Imam et al., 2011; Schlatterer et al., 2011). It is known to be expressed in several cells including fibroblasts, hematopoietic cells, and neurons (Bujor et al., 2011; Artus et al., 2023; Motaln and Rogelj, 2023), and is involved with such functions as cytoskeleton and cell cycle regulation as well as synaptic modeling (Wang, 1993; Woodring et al., 2002; Gutiérrez et al., 2023). Mutations to the SH3 domain of c-Abl are associated with unregulated cell growth and oncogenesis, and in fact c-Abl has been investigated as a contributing factor to chronic myelogenous leukemia (CML) (Bartram et al., 1983; Smith et al., 2003). Regarding NDD pathology, it has been reported that c-Abl activation is involved with cell signaling that regulates neuronal apoptosis in response to Aβ fibrils, suggesting a role for c-Abl in AD pathology (Alvarez et al., 2004). c-Abl activity, which can be regulated in part by oxidative stress, has also been demonstrated to directly interact with α-syn and is associated with α-syn aggregation and dopamine neuron loss in mouse models of PD (Ghosh et al., 2021) and AD (La Barbera et al., 2021). Additionally, lentiviral expression of α-syn has been shown to increased c-Abl activation, resulting in impaired autophagy (Hebron et al., 2013b; Mahul-Mellier et al., 2014). This indicates a synergistic effect between c-Abl activity and disease pathology in PD and suggesting a role for c-Abl in the brain's inability to dispose of toxic proteins in proteinopathies such as AD and PD.

Src, Abl, and Fyn, have also been associated with NDD pathology, including microglial activation, tau phosphorylation, and neurodegeneration, and have been investigated as therapeutic targets (Hebron et al., 2013b; Fowler et al., 2019). Preclinical evidence has shown that treatment with radotinib, an Abl inhibitor, reduces α-syn levels and protects against neurotoxicity in a preformed fibril model of PD (Lee et al., 2018). Similarly, multikinase inhibition with bosutinib, and dual Abl/Src inhibitor, promotes autophagic clearance of Aβ, pTau, and α-syn and reduced markers of inflammation, and is associated with improved behavioral outcomes in animal models of neurodegeneration (Lonskaya et al., 2015; Fowler et al., 2019). Clinical trials (NCT04744532) using bosutinib as a potential therapy for patients with ALS and dementia are currently underway (Mahdavi et al., 2021; Imamura et al., 2022). Sarcatinib, a Src/Fyn inhibitor, has been similarly shown to reduce tau pathology and microglial activation in transgenic AD mice, resulting in improved behavioral outcomes (Kaufman et al., 2015). However, a subsequent Phase II clinical trial in AD patients found that treatment with 100 mg sarcatinib did not slow cognitive decline or loss of brain volume (Van Dyck et al., 2019). Dasatinib, an Abl/Src/c-KIT inhibitor, has shown efficacy in preclinical models at mitigating astrocytic and microglial activation and has been suggested as a potential senolytic therapy (Ryu et al., 2019; Islam et al., 2023). Patients with Chorea-Acanthocytosis treated with dasatinib showed that it was safe and that treatment resulted in stabilization of clinical parameters and increased autophagy markers (Peikert et al., 2021), suggesting its potential as a therapeutic for other NDDs characterized by protein accumulation, including AD and PD.

### 3.5 Platelet derived growth factor receptors

Platelet-derived growth factor receptors (PDGFRs) are a class of RTKs known to regulate cellular proliferation and differentiation and are primarily expressed on fibroblasts, smooth muscle cells, endothelial cells, and neurons (Heldin et al., 1998; Hye-Ryong Shim et al., 2010). Their natural ligands are the platelet-derived growth factors (PDGFs), a family of growth factors associated with angiogenesis, and they can exist in two isoforms, PDGFRα and PDGFRβ and are structurally similar to c-KIT (Heldin and Lennartsson, 2013). Downstream substrates of PDGFRs include PI3K and MAPK, and as such dysregulated PDGFR signaling is associated with certain cancers (Zhang et al., 2007; Li et al., 2012). Increased PDGF levels are also associated with such vascular disorders as atherosclerosis and fibrosis, indicating a potential role for PDGFR signaling in blood vessel related changes observed in such NDDs as AD and PD (Wilcox et al., 1988; Pontén et al., 2003; Smyth et al., 2022). Lastly, PDGFR signaling can activate STATs, while PDGFRβ expression has been shown to regulate inflammation, identifying a means by which PDGFRs may be associated with inflammation in NDDs (Sachsenmaier et al., 1999; He et al., 2015). Studies have found that PDGF and PDGFRβ levels are increased in both the plasma and cerebrospinal fluid (CSF) of AD patients (Sil et al., 2018), suggesting a role for this axis in AD pathogenesis. However, whether these changes are disease-driving or a response to other pathological features remains unclear.

### 3.6 Vascular endothelial growth factor receptors

Vascular endothelial growth factor receptors (VEGFRs) are TKs that serve as growth factor receptors, although VEGFRs have mainly been implicated in the process of angiogenesis (Hicklin and Ellis, 2005; Shibuya, 2014). VEGFRs are primarily expressed on vascular endothelial cells and along lymphatic vessels but have also been shown to play a role in immune cell stimulation and migration (Ohm et al., 2003; Secker and Harvey, 2015; Wheeler et al., 2018). They exist as RTKs at the cell surface and have three subtypes: VEGFR1, VEGFR2, and VEGFR3. Each VEGFR can respond to distinct vascular endothelial growth factors (VEGFs), their endogenous ligands, to promote different cell signaling responses: these include blood vessel permeability, cellular proliferation via PI3K signaling, anti-apoptotic mechanisms, and angiogenesis (Shibuya, 2011). VEGFRs have been shown to be activated in the lower spinal cord of experimental autoimmune encephalomyelitis (EAE) mice that model MS, suggesting a potential role for VEGFRs in MS pathology (Stanojlovic et al., 2016). Additionally, the role of VEGFRs in cellular proliferation has been explored as a possible treatment for various cancers and indicates VEGFRs as potential targets for inducing autophagic clearance of neurotoxic protein aggregates in the proteinopathies (Liu et al., 2017; Liang et al., 2019). Lastly, the critical role VEGFRs play in blood vessel regulation may implicate them in vascular features in diseases such as AD: increased BBB permeability is a common finding in AD brains and has been shown to be predictive of later cognitive decline (Govindpani et al., 2019), suggesting that VEGFR manipulation may serve as a therapeutic medium for alleviated blood vessel-associated damage in AD. Pazopanib is a TKI that primarily inhibits VEGFRs, with cross-reactivity for PDGFRs and c-KIT. Preclinical evidence in tauopathy mice has demonstrated that pazopanib treatment is associated with modulation of astrocyte reactivity and reduction of pTau levels and is associated with behavioral improvements (Javidnia et al., 2017). Interestingly, pazopanib treatment was also associated with increased levels of hippocampal acetylcholine, indicating a method by which VEGFR inhibition may serve to restore normal neurotransmitter balance (Yang et al., 2015).

### 3.7 Colony stimulating factor 1 receptor

Colony stimulating factor 1 receptor (CSF1R) is an RTK primarily expressed on myeloid cells, including microglia, as well as neural progenitor cells and CNS capillary endothelial cells (Nandi et al., 2012; Jin et al., 2014; Hu B. et al., 2021). Its natural ligands are colony stimulating factor 1 (CSF1) and interleukin 34 (IL-34), and its downstream substrates include but are not limited to: Src kinases, PI3K, and STATs (Courtneidge et al., 1993; Novak et al., 1995; Nandi et al., 2012; Stanley and Chitu, 2014). CSF1R signaling is vital for brain development, as CSF1R knockout mice display severe neurodevelopmental deficits including atrophy and functional abnormalities across several brain regions (Hu B. et al., 2021). While important for neural development, the main role for CSF1R signaling appears to be the development and maintenance of microglia, as CSF1R knockout mice exhibit global loss of microglia, indicating the importance of CSF1R to microglial homeostasis (Erblich et al., 2011; Oosterhof et al., 2019). Microglial proliferation and activation are hallmarks of several NDDs, including AD, PD, HD, and ALS, suggesting a potential role by which CSF1R may contribute to neuroinflammation and cognitive decline (Kaur et al., 2019; Clarke and Patani, 2020; Zhu et al., 2022; Wilton et al., 2023; Rim et al., 2024). CSF1R signaling on microglia has been shown to interact with the TREM2 receptor, a protein linked to increased risk for developing AD, indicating a mechanism by which CSF1R may serve as a target for therapeutic intervention in AD (Cheng et al., 2021; Van Lengerich et al., 2023). Additionally, the role of CSF1R in the proliferation of other myeloid cells, such as macrophage (Sehgal et al., 2018), suggest that inhibition of CSF1R may serve to reduce inflammation via peripheral immune cells, which have been demonstrated to infiltrate the brain and cause damage in such disorders as AD, PD, and MS (Høglund, 2014; Zhang X. et al., 2021; Liu et al., 2023). CSF1R inhibitors have been targeted as therapies aimed at reducing microglial-mediated neuroinflammation in NDDs including AD. Treatment with PLX5622, a CSF1R inhibitor used in preclinical models to wipe out microglial populations, has been shown to improve metabolic outcomes in mice (Ali et al., 2020), while treatment with GW2580, another CSF1R inhibitor, was associated with prevention of synaptic degeneration and reduced microglial inflammation (Neal et al., 2020), indicating CSF1R inhibition as a potential medium for alleviating neuroinflammation and NDD pathology. However, further research into disease stage-specific microglial depletion and effective dosing is required before translation of the preclinical findings to human patients. Subsequent investigation into the exact role of CSF1R signaling in microglial-mediated neuroinflammation may yet uncover novel mechanisms by which microglia promote deleterious outcomes in NDD.

### 3.8 Bruton's tyrosine kinase

Bruton's tyrosine kinase (BTK) is a non-receptor tyrosine kinase that is predominantly involved in B cell development and signaling (Khan, 2001). BTK can be activated by upstream substrates including the B cell receptor (BCR) (Takata and Kurosaki, 1996; Petro et al., 2000), toll-like receptors (TLRs) (Jefferies et al., 2003), and Src kinases (Mahajan et al., 1995), and its downstream substrates include phospholipase C-γ (PLCγ) (Takata and Kurosaki, 1996), which can induce transcription of NFAT leading to increased cytokine production (Yablonski et al., 2001; Fric et al., 2012), as well as NFκB (Purvis et al., 2020). This implicates BTK activity in immune cell proliferation and activation, features shown to contribute to disease pathology and worsened cognitive decline in NDDs such as AD, PD, and MS (NCT04338061 and NCT04338022). Specifically with regard to MS, B cell activity has been shown to directly contribute to such pathogenic processes as T cell activation, inflammatory cytokine production, and myelin damage, all of which have been demonstrated to intensify MS pathology (Iglesias et al., 2001; Michel et al., 2015). Subsequent investigations (NCT04032158) have been undertaken aimed at mitigating B cell activity as a means of slowing MS progression and have found that B cell inhibition is sufficient to reduce acute MS flares (Hauser et al., 2008; Reich et al., 2021), further underscoring the potential of BTK inhibition as a means of alleviating inflammation in both MS and other NDDs. Evobrutinib, a selective BTK inhibitor, was shown in a Phase II clinical trial for patients with relapsing MS to decrease the number of enhancing lesions vs. placebo (NCT02975349), although the relapse rate and disability progression did not differ between the two groups (Montalban et al., 2019). Evobrutinib did not meet its clinical endpoints in a subsequent Phase III clinical trial, suggesting that BTK inhibition alone may not be sufficient to alleviate MS progression. Ibrutinib, a brain-penetrant BTK inhibitor originally designed to treat B-cell lymphoma, was administered to 5xFAD and PS19 transgenic mice and was found to suppress Aβ and tau pathology and neuroinflammation, as well as increase the number of dendritic spines on hippocampal neurons, indicating it as a potential treatment for AD patients. Further clinical studies investigating the benefit of ibrutinib in human patients are thus warranted, although treatment has been associated with increased adverse events (Lipsky et al., 2015; Mason et al., 2017; Lee et al., 2021).

## 4 TKIs currently under investigation

TKIs have been heavily exploited as cancer therapeutics (Table 1), primarily due to their role in autophagy, blood vessel remodeling and inflammation. These mechanisms of action overlap between NDDs and cancer and TKIs may at different doses or frequency of administration play a pivotal role in attenuating neurodegenerative processes, including degradation of misfolded or toxic proteins, reduction of inflammation and prevention of fibrotic events of blood vessels in the brain. There are several TKIs in clinical use that are FDA-approved for different uses in oncology or are currently being investigated either for cancer or NDDs. None of these inhibitors (Table 1) are approved for NDDs, as they are poor or not brain penetrant and they do not have the same specificity. In stark contrast with other compounds, KeifeRx is advancing brain-penetrant, kinase targeted and peripherally restricted programs against tyrosine and non-tyrosine kinases (BK40143 and BK40197) that play a role in pathological immune signaling and autophagy (Stevenson et al., 2024).

**Table 1:** TKIs are currently either FDA approved in cancer or under clinical or pre-clinical investigation for NDDs.

**Compounds**	**Mechanisms of action (FDA approval date)**	**Indication/Use**	**Company/route**
Lenvatinib	VEGFR1-3, FGFR1-4, (Smyth et al., 2022), KIT, and RET (2015)	Hepatocellular and (HCC) renal cell carcinoma (RCC)	Esai (oral)
Regorafenib	VEGFR1/Flt1/2/3, PDGFRβ, Kit, RET and Raf-1 (2012)	gastrointestinal stromal tumor (GIST), HCC and RCC	Bayer (oral)
Imatinib	CR/ABL, v-Abl, PDGFR and c-kit (2001)	CML	Novartis (oral)
Cabozantinib	VEGFR2/KDR/Flk-1 and MET, KIT, RET, AXL, TIE2, and FLT3 (2012)	RCC and HCC	Exillisis (oral)
Avapritinib	KIT and PDGFRA, KIT D816V and PDGFRA D842V (2020)	GIST, aggressive systemic mastocystosis (ASM)	Blueprint Medicines Corporation (oral)
Masitinib	c-Kit/c-Abl/PDGFR	Investigational in Phase 2/3 ALS, MS and AD	AB Science (oral)
Ripretinib	KIT and PDGFRA LT3 and VEGFR2/KDR/Flk1 (2020)	GIST	Deciphera Pharmaceuticals (oral)
Dovitinib	Multi-targeted tyrosine kinase (RTK), FLT3, c-Kit, CSF 1R, FGFR1/FGFR3, VEGFR1/Flt-1/VEGFR2/KDR/Flk-1/VEGFR3/Flt 4 and PDGFRα/PDGFRβ	Investigational Phase 2 and 3 (RCC)	Novartis (oral)
Lenvatinib	EGFR1-3, FGFR1-4, PDGFR, KIT, and RET (2015)	RCC, HCC, Thyroid and endometrial	Eisai (oral)
Sitravatinib	VEGFR3/Flt-4, VEGFR2/KDR/Flk-1, VEGFR1/Flt-1, KIT, FLT3, DDR2, DDR1, TRKA, TRKB (2022)	metastatic non-small cell lung cancer	Mirati Therapeutics (oral)
Amuvatinib	Mutant c-Kit, PDGFRα, Flt3, c-Met and c-Ret (2021)	metastatic non-small cell lung cancer (NSCC)	Janssen Biotech (oral)
Bezuclastinib	KIT D816V	Investigational in nonadvanced systemic mastocystosis (NonAdvSM).	Cogent biosciences (oral)
Regorafenib	VEGFR1/Flt-1/2/3, PDGFRβ, Kit, RET and Raf-1 (2012)	Colon and Rectal carcinoma (CRC), GIST and HCC	Bayer (oral)
Vimseltinib	c-FMS (CSF-IR) and c-Kit	Investigational in phase 1 tenosynovial giant cell tumor (TGCT)	Deciphera Pharmaceuticals (oral)
Seralutinib	PDGFRα and PDGFRβ, CSF1R and c-KIT	Investigational in phase 2 pulmonary arterial hypertension.	Gossamer Bio (Inhaler)
Risvodetinib	c-Abl and KIT	Investigational in Phase 1 PD and leukemia	Inhibikase Therapeutics (oral)
Bosutinib	c-Abl and SRC (2012)	Leukemia Investigational in LBD	Pfizer (oral) KeifeRx (oral)
Nilotinib	c-Abl, DDR1, DDR2 (2007)	Leukemia Investigational in AD	Novartis (oral) KeifeRx (oral)
Flumatinib	c-Abl, PDGFRβ and c-Kit	Investigational in CML	Novartis (oral)
Vodobatinib (k0706)	c*-Abl*	Investigational in Phase 2 leukemia and PD and DLB	Sun Pharma Advanced Research Company (oral)
BK40143	c-Abl/c-Kit/Src	Preclinical (AD)	KeifeRx
BK40195	c-Abl/c-Kit/Fyn	Preclinical (PD)	KeifeRx
BK40196	c-Abl/c-kit/Tyro3/PYKEFIVE	Preclinical (FTD)	KeifeRx
BK40197	c-Kit/PYKEFIVE/mTOR	Preclinical (ALS)	KeifeRx

## 5 Discussion

Not only does investigation of the efficacy of novel tyrosine kinase inhibitors in treating NDDs highlight them as therapeutic options for treating NDDs, but it also identifies the potential role of tyrosine kinases is disease pathogenesis. The notable upregulation of TKs in postmortem brains from patients with NDDs suggests their involvement in pathological processes, although their distinct contributions to neurodegeneration have remained unclear. The findings discussed herein implicate them in a host of NDD features, including neurotoxic protein aggregation, microglial-mediated neuroinflammation, vascular fibrosis, and neurotransmitter imbalance, demonstrating the utility of targeting TKs to alter multiple pathological features. One question that remains unanswered is why TKs are upregulated in NDDs brains, and at what point in disease progression this phenomenon becomes prominent; whether increased TK expression precedes clinical effects or is instead a symptom of alternative etiological factors may shed light upon how NDDs arises in the aging brain. TKs have been well classified as important for cellular development and remain vital growth factor receptors in certain scenarios, but it is unknown why they become upregulated in NDDs. One potential hypothesis is that unhealthy brain cells may upregulate certain TKs to increase proliferative signaling and ward off insults as a means of preventing apoptosis in response to such detrimental factors as metabolic dysfunction or inflammation, common features believed to contribute to NDD onset. To this end, data suggests that TKIs serve to undermine this response and instead maintain more normative cellular profiles, promoting autophagic clearance of toxic protein aggregates and inhibiting excessive microglial activation. Future research should be dedicated to exploring age-related changes in TK expression and whether this affects the maximum efficacy of TKIs in promoting cognitive improvement; finding that TKs are expressed in greater numbers only after disease onset would help classify TKIs as disease-modifying treatments, while discovering that TK upregulation precedes pathological findings may suggest their effectiveness at combatting disease pathology before symptom onset in subsets of patients. Another important consideration for TKIs as treatments for NDDs is the potential off-target effects associated with targeting broadly expressed proteins, as well as cross-reactivity when using multikinase inhibitors. Several TKIs have induced adverse events in clinical trials, including arrythmias, weight loss, gastrointestinal irritation, thrombocytopenia, and neutropenia, among others (Shyam Sunder et al., 2023). Considering the potential for adverse events associated with TKI, the usage of TKIs and determination of their effective doses for treating NDDs should closely monitor off-target effects in distal tissues, Binary pharmacology using brain-impenetrant drug antagonists represents one potential method for minimizing off-target effects in the periphery while maximizing CNS penetrance and dosage (Zhang et al., 2022). In addition to the therapeutics mentioned above, dozens of new TKIs are currently being investigated in the drug development pipeline, lending hope that novel effective TKIs to treat NDD may soon be widely available.
